# Surgery Strategies for Gastric Cancer With Liver Metastasis

**DOI:** 10.3389/fonc.2019.01353

**Published:** 2019-12-06

**Authors:** Zai Luo, Zeyin Rong, Chen Huang

**Affiliations:** Department of General Surgery, Shanghai General Hospital, School of Medicine, Shanghai Jiao Tong University, Shanghai, China

**Keywords:** gastric cancer, liver metastasis, surgery, strategy, R0 resection

## Abstract

Gastric cancer with liver metastasis is defined as advanced gastric cancer and remains one of the deadliest diseases with poor prognosis. Approximately 4–14% of patients with gastric cancers presented with liver metastases at the initial diagnosis. Owing to its incurability, first-line treatment for gastric cancer with liver metastases is systematic chemotherapy, whereas surgery is usually performed to alleviate severe gastrointestinal symptoms. However, continuously emerging retrospective studies confirmed the role of surgery in gastric cancer with liver metastases and showed significantly improved survival rate in patients assigned to a group of surgery with or without chemotherapy. Therefore, more and more convincing data that resulted from prospective randomized clinical trials is in need to clarify the surgery strategies in patients with gastric cancer with liver metastasis.

## Introduction

Gastric cancer (GC), as the third most frequent cause of cancer-related death for human cancers in the world, continues to carry a noticeably higher fatality-to-case ratio, accounting for exceeding 782,000 confirmed cases died in 2018 worldwide (1). Especially in China, based on data from National Central Cancer Registry of China (NCCR) in 2015, gastric cancer was the most common cancer and the leading cause of cancer death except for lung cancer (2). Predominantly due to late-onset and non-specific symptoms and lack of active screening programs, ~34% of patients have distant metastases according to the Surveillance, Epidemiology, and End Results (SEER) Database (3), and nearly 4–14% of patients present with liver metastases at the initial presentation (4). In fact, the leading causes of death for gastric cancer include local recurrence, gross peritoneal dissemination, direct invasion to other organs, and extensive distant organ metastases. Anatomically speaking, the liver is the most common site of hematogenous metastases for advanced gastric cancer. Gastric cancer with liver metastases (GCLM) is generally classified into two types: one is synchronous metastases, which defined as metastases occurring before or during surgery or within 6 months after gastrectomy, and the other is metachronous metastases, which defined as metastases identified at least 6 months after gastrectomy (5). Synchronous GCLM is detected in nearly 5–10% of gastric cancer patients at diagnosis (6), whereas metachronous GCLM is in up to 37% after “curative” resection of primary gastric cancer (7).

According to practical clinical guidelines, such as the National Comprehensive Cancer Network (NCCN), GCLM was regarded as stage IVb disease and unresectable tumor, which not only showed aggressive oncological behavior but also accompanied by distant metastases. And it was traditionally recommended with systemic chemotherapy including CF (cisplatin and fluorouracil) or ECF (epirubicin, cisplatin, and fluorouracil) chemotherapeutic regimens (8). Recently, accumulating clinical trials have achieved significant progress in chemotherapy. For example, for HER2-negative advanced gastric cancer, the findings of the SPIRITS trial revealed the superiority of S-1 plus cisplatin to S-1 alone in advanced gastric cancer (9). Furthermore, results of the G-SOX trial found that S-1 plus oxaliplatin was non-inferior to S-1 plus cisplatin in advanced gastric cancer, mainly in less toxic and more convenient clinically (10). For HER2-positive advanced gastric cancer, discoveries of the ToGA trial found that chemotherapy regimen consisting of capecitabine plus or fluorouracil plus cisplatin in combination with trastuzumab was a promising option for patients with HER2-positive advanced gastric cancer (11). In addition, results of the ATTRACTION-2 trial indicated the survival benefits of nivolumab in patients with advanced gastric or gastroesophageal junction cancer (12). Although major progress was made in chemotherapy and molecularly targeted biological therapy (13, 14), until now, the median survival time (MST) of patients with GCLM was between 7 and 14.1 months (9–12). Given the dismal prognosis of patients with GCLM, there was an urgent need to develop better treatment strategies for GCLM in the absence of institutional guidelines or protocols.

Inspired by substantial survival benefits and compelling evidence of surgery in patients with colorectal cancer liver metastases (15, 16), many clinical surgeons explored the role of surgery in GCLM, which was considered as a crucial intervention and the most essential step to cure disease and to prolong patient life (17). Increasing systemic and aggressive oncological behaviors were shown in GCLM (18), compared with colorectal cancer liver metastases; gastrectomy was reserved for the palliation of severe gastrointestinal symptoms such as refractory hemorrhage and obstruction in patients with GCLM based on NCCN (8). On the contrary, the Guidelines Committee of the Japan Gastric Cancer Association was in favor of surgical resection of potentially resectable M1 disease (19), and recent studies showed that the potential of surgical resection in selected GCLM, which can bring MST between 9 and 67.5 months and 5-year survival, varies from 0 to 42%, inspiringly (5, 20, 21). This review aims to summarize recent studies underpinning the surgical resection for GCLM and to explain the surgery strategies in different clinical classifications of GCLM.

## Current Evidence

### Controversies in the Surgical Resection of Gastric Cancer With Liver Metastasis

From the perspective of the routine clinical application, objective assessments of clinical data about surgical resection in GCLM are essential to investigate the surgery strategies in GCLM. Although surgery is recommended to alleviate severe gastrointestinal symptoms in consensus, the utility of surgery in GCLM still remains highly controversial. More recently, inconsistent and contradictory findings of surgical resection in GCLM have emerged in published literature (22, 23).

A public clinical trial (REGATTA) (22) failed to improve the overall survival (OS) rate in advanced gastric cancer patients assigned to gastrectomy plus post-operative chemotherapy than in those assigned to chemotherapy alone (14.3 vs. 16.6 months). However, evidence from a clinical trial (AIO-FLOT3) (23) showed different outcomes. Compared with patients who experienced chemotherapy alone, patients who experienced neoadjuvant chemotherapy followed by surgical resection had superior OS (22.9 vs. 10.7 months). Notably, the design of the REGATTA trial differed from the design of the AIO-FLOT3 trial in some respects, which possibly had influenced outcomes of the trial. First, most GCLM patients enrolled in the REGATTA trial were accompanied by peritoneal metastases, who were recognized as the worst kind in advanced GC patients in prognosis. Second, the surgical management in the REGATTA trial was restricted to D1 lymphadenectomy only, whereas in the AIO-FLOT3 trial, the surgical management adopted gastrectomy with D2 lymphadenectomy, which was recommended for total or subtotal distal gastrectomy (24). Third, compared with gastrectomy plus chemotherapy adopted in the REGATTA trial, the AIO-FLOT3 trial utilized neoadjuvant chemotherapy followed by surgical resection in the treatment plan. Collectively, the above evidence reveals the crucial factors including patient selection, surgical procedures, and treatment options in a multimodality approach to GCLM.

### Potential Superiority of Surgery in Gastric Cancer With Liver Metastasis

Available evidence of surgery for patients with GCLM mostly relies on retrospective studies, systematic reviews, and prospective trials. Data published after 2000 mostly showed significant and prognostic benefits of surgical resection for GCLM (Table 1) (5, 6, 20, 21, 25, 73), and the benefits were in continuous increase owing to advancements in accurate diagnosis, patient selection, perioperative nutritional support, anesthetic techniques, surgery approaches, management of post-operative complications, and enhanced recovery after surgery.

**Table 1:** Demographics and survival in GCLM patients underwent surgical resection.

**References**	**Year**	**Country**	**Type**	**Study** **interval**	**No. of** **Patients**	**Median** **Age**	**Post-operative 30-day mortality (%)**	**Overall survival**			
								**1 year (%)**	**3 years (%)**	**5 years (%)**	**MST (months)**
Adam et al. (25)	2006	France	Retro	1983–2004	64	NR	NR	NR	NR	27	15
Aizawa et al. (26)	2014	Japan	Retro	1997–2010	53	66	NR	NR	NR	18.6	27.4
Ambiru et al. (27)	2001	Japan	Retro	1975–1999	40	63	0	NR	NR	18	12
Baek et al. (28)	2013	Korea	Retro	2003–2010	12	61	0	65	NR	39	31
Chen et al. (29)	2013	China	Retro	2007–2012	20	57	0	NR	NR	15	22.3
Cheon et al. (30)	2008	Korea	Retro	1995–2005	41	60	1.72	75	32	21	17
Choi et al. (31)	2010	Korea	Retro	1986–2007	14	65	NR	67	38.3	NR	NR
Dittmar et al. (32)	2012	Germany	Retro	1995–2009	15	57	0	NR	NR	27	48
Fukami et al. (33)	2017	Japan	Retro	2001–2012	14	66	NR	71.4	42.9	42.9	27.9
Fuji et al. (34)	2001	Japan	Retro	1979–1999	10	58.5	10	60	20	10	NR
Garancini et al. (35)	2012	Italy	Retro	1998–2007	21	64	0	68	31	19	11
Guner et al. (36)	2016	Korea	Retro	1998–2013	68	61	NR	79.1	40.6	30	NR
Hirai et al. (37)	2006	Japan	Retro	1993–2004	14	NR	NR	NR	NR	41.6	NR
Hwang et al. (38)	2009	Korea	Retro	1995–2005	73	59	NR	NR	NR	NR	20
Imanura et al. (39)	2001	Japan	Retro	1990–1997	17	NA	NR	60	25	NR	NR
Kinoshita et al. (40)	2015	Japan	Retro	1990–2010	256	64	NR	77.3	41.9	31.1	31.1
Koga et al. (41)	2007	Japan	Retro	1985–2005	42	64	0	76	48	42	34
Kokkola et al. (42)	2012	Finland	Retro	2000–2009	23	61.4	NR	NR	NR	NR	14.3
Komeda et al. (43)	2014	Japan	Retro	2000–2012	24	69.5	0	78.3	40.1	40.1	22.3
Lee et al. (20)	2017	Korea	Retro	2000–2014	7	59.2	NR	NR	NR	68.6	67.5
Li et al. (6)	2015	China	Retro	2008–2011	25	61.4	NR	72	NR	NR	20.5
Li et al. (44)	2017	China	Retro	1996–2012	34	62	NR	73.5	36.9	24.5	26.2
Liu et al. (45)	2012	China	Retro	1995–2010	35	NR	NR	58.1	21.7	NR	15
Liu et al. (46)	2015	China	Retro	1990–2009	35	56	0	NR	NR	14.3	33
Makino et al. (47)	2010	Japan	Retro	1992–2007	16	NA	0	82.3	46.4	37.1	31.2
Markar et al. (48)	2016	UK	Retro	1997–2012	78	NR	7.2	64.1	NR	38.5	NR
Miki et al. (49)	2012	Japan	Retro	1995–2009	25	72	NR	73.9	42.8	36.7	33.4
Morise et al. (50)	2008	Japan	Retro	1989–2004	18	64	NR	56.3	27.3	27.3	13
Nishi et al. (51)	2018	Japan	Retro	1996–2008	39	64	0	56.4	17.9	10.3	14
Nomura et al. (52)	2009	Japan	Retro	1991–2005	17	65.8	NR	NR	NR	30.8	21
Ohkura et al. (53)	2015	Japan	Retro	1985–2014	9	66	NR	88.9	29.6	NR	NR
Okano et al. (54)	2002	Japan	Retro	1986–1999	19	69	NR	77	34	34	21
Oki et al. (55)	2016	Japan	Retro	2000–2010	94	70	NR	86.5	51.4	42.1	40.8
Qiu et al. (56)	2013	China	Retro	1998–2009	25	NR	0	96	70.4	29.4	38
Roh et al. (et al. (57)	2005	Korea	Retro	1988–1996	11	61	0	73	NR	27	19
Rudloff et al. (58)	2014	USA	Pro	2009–2012	9	45	NR	44.4	33.3	22.2	11.3
Ryu et al. (59)	2017	Japan	Retro	1997–2015	14	NR	NR	84.6	51.3	51.3	NR
Saiura et al. (60)	2002	Japan	Retro	1981–1998	10	60.5	30	50	30	20	25
Sakamoto et al. (61)	2007	Japan	Retro	1990–2005	37	64	0	NR	NR	11	31
Schildberg et al. (62)	2012	Germany	Retro	1972–2008	31	65	6	NR	NR	13	NR
Shinohara et al. (63)	2015	Japan	Retro	1995–2010	22	NR	0	86	26	26	22
Shirabe et al. (64)	2003	Japan	Retro	1979–2001	36	66	0	64	26	26	NR
Song et al. (65)	2017	China	Retro	2001–2012	96	63	0	87.5	47.6	21.7	34
Takemura et al. (66)	2012	Japan	Retro	1993–2011	64	65	0	84	50	37	34
Thelen et al. (5)	2008	Germany	Retro	1988–2002	24	64	4.2	38	16	10	9
Tiberio et al. (67)	2016	Italy	Retro	1990–2013	105	68	0.9	58.2	20.3	13.1	14.6
Tsujimoto et al. (68)	2010	Japan	Retro	1980–2007	17	66	NR	75	37.5	31.5	34
Turanli et al. (21)	2010	Turkey	Pro	2005–2008	18	NR	NR	NR	0	0	14.1
Ueda et al. (69)	2009	Japan	Retro	1991–2005	15	NR	0	80	NR	60	13.4
Viganò et al. (70)	2013	Italy	Retro	1997–2008	20	61.5	0	95	63.2	33.2	52.3
Wang et al. (71)	2012	China	Retro	2003–2008	30	60	0	43.3	16.7	16.7	11
Wang et al. (72)	2014	China	Retro	1996–2008	39	64	0	56	17.9	10.3	14
Zacherl et al. (73)	2002	Austria	Retro	1980–1999	15	62	0	36	14.3	0	8.8

*Retro indicates retrospective study; NR, not reported; MST, median survival time*.

Recently, principally from East Asia and Europe, large retrospective studies on the surgical resection of GCLM have shown continuously acceptable survival outcomes for selected patients. Nishi et al. (51) demonstrated that the overall 1- and 3-year survival rates after hepatic resection for GCLM were 88.9 and 17.8% in 10 selected patients, respectively, with an MST of 21.5 months and no post-operative mortality. Similarly, in a retrospective single-center study involving 34 patients with GCLM, Ryu et al. (59) investigated the significance of surgical procedures including hepatic resection for more massive metastases and surgical microwave ablation for patients who had a high operative risk and identified prognostic factors. The results showed acceptable morbidity and favorable long-term outcomes, as the 1-, 3-, and 5-year OS rates after surgery were 86.5, 51.4, and 42.1%, respectively, and the 1-, 3-, and 5-year recurrence-free survival (RFS) rates were 38.5, 28.0, and 28.0%, respectively, with no significant survival differences for varied surgical treatments (*P* = 0.213).

Meanwhile, a nationwide retrospective study from England also showed that gastrectomy combined with hepatectomy for synchronous GCLM might carry survival benefits in selected patients (48). Kaplan–Meier curve analyses showed that patients who were selected to have gastrectomy with additional hepatectomy for liver metastases (GGH group) had survival similar to that of patients who had gastrectomy in the absence of liver metastases (GG group) (*P* = 0.196) and improved survival than did patients who had gastrectomy without liver resection for liver metastases (GGNH group) (*P* < 0.001) and patients with GCLM who had no surgery (GNS group) (*P* < 0.001). As for mortality, the GGH group and GGNH group had similar 30-day mortality (*P* = 0.246), whereas the former had significantly improved 90-day mortality (*P* = 0.009), 1-year mortality (*P* < 0.001), and 5-year mortality (*P* < 0.001); and the GNS group had the worst OS and highest mortality at 30, 90 days, 1, and 5 years (*P* < 0.001) in the four groups. The results of this study revealed that gastrectomy combined with additional surgical resection of liver metastases was better than palliative treatment or gastrectomy without resection of liver metastases for patients with GCLM in survival benefits.

To reassess this bias problem in full measure, many systematic reviews and pooled analyses were conducted. A systematic review launched by Liao et al. (74) included eight non-randomized studies, representing a total of 677 patients with GCLM. The median OS time in patients who underwent gastrectomy combined with hepatectomy was significantly prolonged, as compared with the median OS time of those who underwent palliative therapy (23.7 vs. 7.6 months), with survival rates of the two arms of 69, 40, 33%, and 27, 8, 4% at 1, 2, and 3 years, respectively. Compared with palliative therapy, hepatectomy was associated with significantly lower mortality at 1-year (OR 0.17, *P* < 0.001) and 2-year (OR 0.15, *P* < 0.001). Owing to the disparity in the stage of disease, differences of the regimen of chemotherapy, and preference of surgery of surgeons (75), patients who underwent hepatectomy in Western countries showed lower median rates of OS at 1 year (60 vs. 76%), 2 years (30 vs. 47%), and 3 years (23 vs. 39%) than did those in Asian countries.

As previously stated, most of the published papers on surgical resection in patients with GCLM came from retrospective data, whereas only four randomized controlled trials (RCTs) investigated the role of surgery for patients with GCLM so far. The REGATTA trial was the first RCT to compare gastrectomy followed by chemotherapy with chemotherapy alone concerning OS in patients with GCLM (22). Findings from this trial denied the survival efficacy of palliative gastrectomy followed by chemotherapy from an interim analysis, which had caused the interruption of this trial in 2016. However, to some extent, results from the AIO-FLOT3 trial countered those of the REGATTA trial by strict inclusion criteria, surgical approaches, and treatment regimens (23). The AIO-FLOT3 trial exhibited favorable survival in patients with GCLM who received neoadjuvant chemotherapy and later underwent surgical resection, which had provided a rationale for the ongoing AIO-FLOT5 trial (NCT02578368) (76). Compared with the REGATTA trial, the AIO-FLOT5 trial excludes the enrollment of patients with clinically visible tumors of the peritoneum and >P1 peritoneal tumors, adopts a complete resection of a primary tumor including standardized lymphadenectomy (R0 and at least D2), and adjusts the place of chemotherapy and surgery. Hopefully, if this trial was proved to be effective, it could potentially lead to a new standard of therapy. Another ongoing trial named SURGIGAST (NCT03042169), which has not recruited patients, aims to compare the OS of palliative surgical resection plus chemotherapy with that of chemotherapy alone for stage IV gastric cancer including GCLM (77).

Despite the significant survival benefits from gastrectomy combined with hepatectomy over non-resectional management in patients with GCLM, as well as favorable published outcomes from chemotherapy followed by surgery over chemotherapy alone, it must be stressed that most of data came from retrospective studies and systematic reviews. Thus, outcome data from the AIO-FLOT5 trial and the SURGIGAST trial are awaited to verify the survival benefit of surgical resection suggested by retrospective studies and systematic reviews.

### Prognostic Factors and Patient Selection in Gastric Cancer With Liver Metastasis

A considerable amount of published literature about surgery in GCLM illustrates the ascendency of surgery. However, it is conspicuous that not every patient will benefit from surgery. Hence, prognostic evaluation is crucial to identify the suitable candidates for radical surgery, which are of gastric cancer, liver metastases (synchronous disease), and liver metastases alone (metachronous disease), from those who will not benefit from surgery.

Lately, in a multicenter retrospective study, Tiberio et al. (78) compared the application of radical surgery vs. palliative gastrectomy or palliative surgery without resection in GCLM, in which radical surgery had achieved better long-term results than others in the 5-year survival rate (9.3, 2.1, and 0%, respectively). In light of this, they further recognized the best candidates for radical surgery through systematically investigating the patient-related, gastric cancer-related, metastasis-related, and treatment-related prognostic factors. Results confirmed that the invasive depth of primary tumor (*P* < 0.001), curative surgical procedure (R0 resection; *P* = 0.001), timing of hepatic involvement (*P* < 0.001), and adjuvant chemotherapy (*P* < 0.001) were associated with long-term survival, independently. Especially in R0 resection, results implied that it can significantly reduce the possibility of recurrence in GC patients with liver oligometastasis, even in patients with multiply scattered metastases in both lobes of the liver.

Accordingly, in the metachronous disease, Tiberio et al. (79) also revealed that T4 gastric cancer (*P* = 0.019), the presence of lymph node metastases (*P* = 0.05), and grade 3 GC (*P* = 0.018) displayed negative prognostic factors. Moreover, a multivariate analysis demonstrated that a therapeutic strategy of liver metastases was highly associated with survival as well, in particular when R0 resection was performed (*P* < 0.001).

Likewise, based on real-world data, the AGAMENON registry involving 1,792 patients with advanced GC (80), distal esophagus, or gastroesophageal junction revealed higher 3-year survival rate after metastasectomy than non-metastasectomy (30.6 vs. 8.4%; *P* < 0.001) and median OS since metastasectomy of 16.7 months. With the use of a state-arrival extended Markov proportional hazard (PH) model, a multivariate analysis indicated the presence of a HER2-positive tumor treated with trastuzumab (*P* = 0.001) and chemotherapy followed by surgical procedure (*P* < 0.001) as favorable predictors of survival. Moreover, they also found that the unreasonable interval time between the initiation of chemotherapy and surgery appeared to worsen outcomes. Their results also recommended that 5 months as interval time benefits most patients, which is consistent with the AIO-FLOT3 trial.

Also, Takemura et al. (66) reported the overall 5-year survival rate of 37% and the MST of 34 months in 64 patients achieved macroscopically complete (R0 or R1) resections. Among 64 patients, 50 patients had the largest hepatic metastasis of more than 5 cm in diameter, and 14 patients had <5 cm in diameter (*P* = 0.07). Results demonstrated that patients with a maximum diameter of hepatic metastasis >5 cm had poorer long-term survival (*P* = 0.018).

Above all, most of identified prognostic factors were similar with those in various literature through multivariate analyses, which could be roughly divided into five major categories that consisted of primary tumor-associated, liver metastasis-associated, extrahepatic metastasis-associated, and treatment-associated prognostic factors and others, as shown in Table 2. However, these factors were mainly identified from retrospective studies in single center or multicenter, which need to be validated in prospective clinical studies to further confirm their prognostic role.

**Table 2:** Independent favorable prognostic factors for surgery in patients with GCLM.

**Categories**	**Favorable prognostic factors**	**References**
Primary tumor	No serosal invasion	(40, 66)
	Lower T stage	(33, 49, 65, 67, 78, 79)
	No lymphatic or venous invasion	(46, 55, 72, 79)
Liver metastases	Unilobar involvement	(26, 47, 61, 73, 78, 81)
	Number of metastatic lesions ≤ 3, especially for solitary metastasis	(30, 35, 40, 41, 46, 52–56, 61–63, 65, 69–72)
	Diameter of greatest lesion ≤ 5 cm	(36, 40, 43, 53, 66, 68)
	Metachronous metastases	(27, 51, 54, 62, 67)
Extrahepatic metastasis	Absence of peritoneal metastasis	(38, 39, 44, 69, 71)
Treatment	Negative margin (R0)	(5, 21, 30, 35, 62, 67, 69, 82)
	D2 Lymphadenectomy	(68)
	Neoadjuvant chemotherapy	(23)
	Post-operative chemotherapy	(42, 56, 67, 78)
	Response to chemotherapy	(6, 70)
Other	Lower CEA and CA 19-9 levels	(33, 59)
	HER2-positive tumor treated with trastuzumab	(23, 80)

*GCLM, gastric cancer with liver metastases; CEA, carcinoembryonic antigen; CA 19-9, carbohydrate antigen 19-9*.

## Surgery in Different Categories of Gastric Cancer With Liver Metastasis

### New Classified Evaluation for Gastric Cancer With Liver Metastasis

Although the Lauren classification and the WHO classification are popular in pathological grading of GCs, they are insufficient to guide personalized treatments, especially in GCLM. New classified evaluation for GCLM is thus required. Encouragingly, recent advancements in retrospective studies and prospective studies have greatly facilitated the identification of potential candidates.

**Figure 1:**
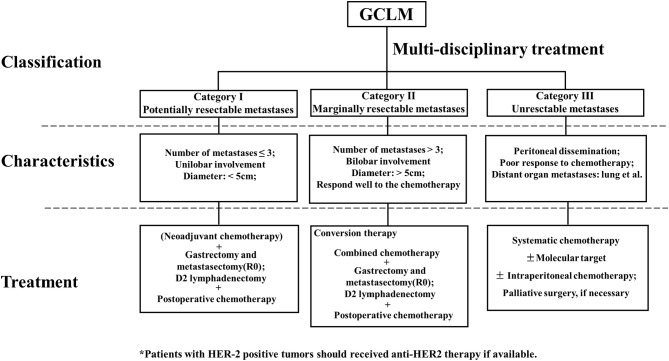
Clinical classification, characteristics, and treatment of gastric cancer with liver metastases (GCLM).

Referring to the clinical study on GCLM and classification of stage IV GC (83), we divided GCLM patients into three categories, as shown in Figure 1. First, GCLM could be divided into the potentially resectable tumor (category I), marginally resectable tumor (category II), and unresectable tumor (category III) according to the analysis of clinical decision making in multidisciplinary treatment. For example, macroscopic peritoneal dissemination was considered an essential factor during the classification process, because patients with peritoneal dissemination or positive peritoneal cytology had significantly poor prognosis (84). Second, patients of category I were recommended to undergo surgery followed by post-operative chemotherapy or to receive neoadjuvant chemotherapy combined with surgery. Patients in category II were suggested to adopt conversion therapy aimed to an R0 resection after combined chemotherapy. Patients in category III, who also had obstruction and bleeding of the gastrointestinal tract in some cases, were advised to receive palliative chemotherapy.

### Surgery Strategies in Different Categories of Gastric Cancer With Liver Metastasis

#### Surgery in Resectable Liver Metastases (Category I)

Potentially resectable liver metastases (category I) were characterized by <5 metastasis (better for solitary metastasis), with the diameter of the largest metastatic lesion measuring <5 cm and metastasis occurring in one liver lobe, which was regarded as a technically resectable metastasis.

For patients who conformed to the defining characteristics of category I, evidence from clinical trials and retrospective studies recommended them to undergo neoadjuvant chemotherapy followed by R0 resection of hepatic metastasis with or without primary GC and D2 lymphadenectomy and post-operative chemotherapy. Komeda et al. (43) indicated that the overall 5-year survival rate and MST of patients with GCLM who underwent gastrectomy followed by curative hepatectomy were 40.1% and 22.3 months, respectively. Especially in patients with a maximum size of liver metastasis ≤ 5 cm, they had higher overall 5-year survival than had patients with a maximum size of liver metastasis > 5 cm (51.7 vs. 14.3%). Furthermore, in a retrospective study that enrolled 24 patients with GC with two or three liver-limited metastases, Shirasu et al. (81) found no survival benefit for patients who experienced hepatectomy only compared with chemotherapy only (*P* = 0.146). However, recurrence and death occurred in none of the patients who received initial chemotherapy followed by surgery. Despite small sample size of patients, this study still should be regarded as a direction for further study.

Similarly, in a prospectively comparative study involving 49 patients with synchronous GCLM, Li et al. (6) compared patients assigned to R0 resection of primary tumor and liver metastasis as well as D2 lymphadenectomy followed by post-operative chemotherapy with patients assigned to chemotherapy only. Results revealed that the MST of surgery group was significantly longer than that of the control group (20.5 vs. 9.1 months). Moreover, the response to chemotherapy was indicated by the prognostic factors only through their multivariate analysis. Remarkably, the AIO-FLOT3 trial (23) enrolled 60 patients with liver metastases of <5 to receive eight cycles of the FOLT (fluorouracil, oxaliplatin, leucovorin, and docetaxel) chemotherapy in total, 36 of whom underwent surgery to achieve margin-free (R0) resection after the first four cycles of neoadjuvant chemotherapy. Compared with 24 patients assigned to chemotherapy only, 36 patients with surgery had more favorable MST (31.3 vs. 15.9 months) and progression-free survival (26.7 vs. 8.4 months).

In this case, initial gastrectomy and hepatectomy aimed to achieve R0 resection; otherwise, it should combined with neoadjuvant chemotherapy. Indeed, R0 resection was a microscopically margin-negative resection, in which no gross or microscopic tumor was kept in the primary tumor site, which could remove the tumor and retain tissues to the hilt. Simultaneously, neoadjuvant chemotherapy was able to treat micrometastases at an early stage to downstage the primary tumor and obtained a higher R0 resection rate. Moreover, post-operative chemotherapy acted as a “supervisor” to maintain the state of R0 resection, for prevention of progression and recurrence of metastasis of gastric cancer. Thus, patients in this category were highly inclined to achieve R0 resection and obtain reduced recurrence rate.

#### Surgery in Marginally Resectable Liver Metastases (Category II)

Marginally resectable liver metastases (category II) was composed of patients with multiple liver metastases (>3), maximum tumor diameter that exceeds 5 cm, or bilobar invasion with the absence of peritoneal metastases. This category was regarded as oncologically and technically unresectable.

In clinical practice, surgery is controversial for these patients, as they are usually offered chemotherapy. However, existing evidence indicated that initially marginally resectable and unresectable gastric cancer could be converted into resectable gastric cancer by novel combined chemotherapy (83–85). Thus, recent studies begin to focus on surgery with an expectation of R0 resection performed in originally unresectable and marginally resectable GCLM that responded to chemotherapy. Fukuchi et al. (84) selected S-1 plus cisplatin or paclitaxel as initial combination chemotherapy for advanced gastric cancer including GCLM. Compared with patients who only received chemotherapy, patients treated with chemotherapy plus surgery had a prolonged survival at 5 years (43 vs. 1%).

Moreover, among patients who underwent conversion therapy, patients who underwent R0 resection had significantly more favorable survival as opposed to those who underwent R+ resections (49 vs. 15% in a 5-year survival rate). In a recent retrospective study involving patients with marginally resectable tumor, Yamaguchi et al. (82) reported that the MST of patients assigned to conversion therapy was 30.5 months, whereas that of patients assigned chemotherapy alone was 11.0 months (*P* < 0.05). In a group of conversion therapy, patients underwent R0 resection had prolonged survival time than had those who underwent R+ resection (56.2 vs. 16.3 months).

In spite of the encouraging outcomes mentioned, the limitations of the above studies should be noted. First, enrolled patients for most studies have experienced for a long period lack of consistencies in the decision making of diagnosis and in approaches of chemotherapeutic regimen and surgery. Second, inherent selection bias occurred in retrospective data including response to chemotherapy and performance status, which could affect outcomes. Third, in almost all retrospective studies, owing to the insufficiency in evidence of clinical characteristics including laboratory data and molecular classification, clinicopathological factors and response to chemotherapy were considered as major factors to predict the candidates for potential R0 surgical resection. Consequently, existing studies should accelerate the implementation of randomized clinical trials to determine the role of conversion therapy and to explore the effect of laboratory data and molecular classification on survival benefit to provide a guideline for patient stratification and personalized treatment in GCLM (86).

#### Surgery in Unresectable Liver Metastases (Category III)

Unresectable liver metastases (category III) contained patients with macroscopically peritoneal dissemination or extensive metastases in multiple organs, who carry a worse or less favorable prognosis.

According to recent studies, patients in category III could benefit from conversion therapy as well. However, only a small fraction of patients who responded well to chemotherapy were accessible to achieve R0 resection (87). Moreover, palliative chemotherapy remained as a mainstream treatment according to clinical guidelines. Consistent with palliative radiotherapy (88), palliative surgery also plays a vital role in coping with obstruction and bleeding of the gastrointestinal tract.

Above all, because most evidence came from retrospective studies, defining the role of surgery in different categories of GCLM was in need of more robust evidence from prospective randomized clinical trials. Furthermore, a combination of clinical classification and molecular classification of GCLM might accelerate the identification of novel therapeutic targets and formation of personalized treatment (89, 90).

## Perspectives

In summary, despite the increasing evidence in favor of surgery in GCLM, the indication and extent of surgery, including the selection of patients and the potential to achieve R0 resection, should be carefully discussed and determined. Emerging research indicated that hepatic arterial infusion chemotherapy (HAIC), radiation therapy, and radiofrequency ablation (RFA) provided alternative treatment modalities for GCLM (36, 91, 92). Importantly, prospective randomized clinical trials are needed urgently to clarify the indication and the surgery strategies in GCLM.

## Author Contributions

ZL and CH were involved in the concept and design. ZL, ZR, and CH wrote, reviewed, and revised the manuscript. CH supervised the manuscript.

### Conflict of Interest

The authors declare that the research was conducted in the absence of any commercial or financial relationships that could be construed as a potential conflict of interest.
